# Permanent Magnet Tracking Method Resistant to Background Magnetic Field for Assessing Jaw Movement in Wearable Devices

**DOI:** 10.3390/s22030971

**Published:** 2022-01-26

**Authors:** Mantas Jucevičius, Rimantas Ožiūnas, Gintautas Narvydas, Darius Jegelevičius

**Affiliations:** 1 Biomedical Engineering Institute, Kaunas University of Technology, K. Baršausko g. 59, LT-51423 Kaunas, Lithuania; darius.jegelevicius@ktu.lt; 2 Investigo, UAB, Draugystės g. 17-1, LT-51229 Kaunas, Lithuania; info@investigo.lt; 3 Faculty of Electrical and Electronics Engineering, Kaunas University of Technology, Studentų g. 50, LT-51368 Kaunas, Lithuania; gintautas.narvydas@ktu.lt

**Keywords:** bruxism evaluation, jaw position detection, masticatory trajectory, 3D motion tracking

## Abstract

There is a large gap between primitive bruxism detectors and sophisticated clinical machines for jaw kinematics evaluation. Large, expensive clinical appliances can precisely record jaw motion, but completely restrain the patient for the duration of the test. Wearable bruxism detectors allow continuously counting and recording bites, but provide no information about jaw movement trajectories. Previously, we developed a permanent magnet and three-axis magnetometer-based method for wearable, intra-oral continuous jaw position registration. In this work, we present an effective solution of the two main drawbacks of the method. Firstly, a two-adjacent-magnetometer approach is able to compensate for background magnetic fields with no reference sensor outside of the system’s magnetic field. Secondly, jaw rotational angles were included in the position calculations, by applying trigonometric equations that link the translation of the jaw to its rotation. This way, we were able to use a three-degree-of-freedom (3-DOF) magnetic position determination method to track the positions of the 5-DOF human masticatory system. To validate the method, finite element modeling and a 6-DOF robotic arm (0.01 mm, 0.01°) were used, which showed a 37% decrease in error in the average RMSE = 0.17 mm. The method’s potentially can be utilized in small-scale, low-power, wearable intra-oral devices for continuous jaw motion recording.

## 1. Introduction

A method for intra-oral 24 h continuous jaw position tracking could open up unprecedented opportunities in jaw research. Current solutions for precise jaw kinematics evaluation are external and stationary appliances that are unfit for continuous use [1,2,3]. Moreover, such a method would be very perspective in bruxism diagnostics. Based on the severity of the condition, 8–31% of the population has this disorder [4]. Undiagnosed, it can result in teeth attrition, cracks in the enamel, or implant failure [5]. Available devices for bruxism diagnostics are either based on masseter muscle EMG [6] or occlusal splints with integrated pressure sensors [7]. Such devices are suitable for confirming the condition and providing biofeedback for damage prevention. However, they are unable to provide any information about jaw movements and trajectories. To fill the gap between primitive mastication detectors and sophisticated clinical appliances, a magnetic method for intra-oral jaw position determination was proposed in our previous research [8]. The method was able to determine the positions of the test trajectory with an average RMSE of 0.260 mm in laboratory experiments [8]. However, it can only determine linear position displacements on three axes, resulting in a three-degree-of-freedom (DOF) solution. Therefore, in this work, we propose trigonometric equations to relate jaw linear displacements to jaw rotational angles, which could significantly enhance the algorithm.

**Figure 1:**
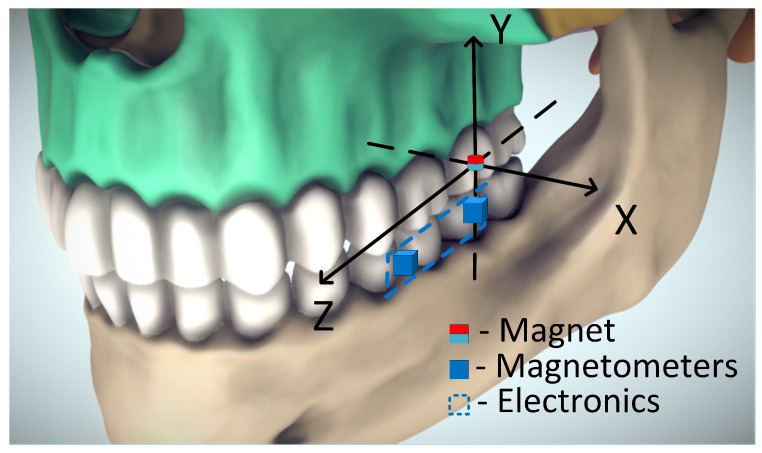
Illustration of the concept of the method. Based on [11].

The second drawback of the previously proposed method is a direct background magnetic field (BMF) compensation, which is based on subtraction. In the direct BMF compensation method, a reference magnetometer has to be placed outside of the magnetic field of the permanent magnet that is being measured. Recorded reference values are then subtracted from the main sensor data. The subtraction method has been described in detail by Ryoo et al. to compensate the BMF for the main magnetometer of an autonomous land vehicle, guided by magnetic markers embedded in the road [9]. In our previous work, for the case of a (2 × 2 mm) cylindrical magnet with 1.4 T residual magnetism, we chose to place the reference sensor at 35 mm away. This was chosen as the minimal distance for the subtraction method to remain useful and fit intra-orally, with a 7 μT offset remaining of the average 35 μT ambient field [8]. However, for full subtractive compensation, the reference magnetometer should be completely outside of the field of the permanent magnet. Another way to mitigate the BMF is by magnetic shielding, which requires covering the volume of the measurement with a ferromagnetic material that has a high permeability. There are effective and commercially available iron and nickel alloys designed for this purpose, such as Mu-metal [10]. However, such an approach is unfit for wearable biomedical applications. We aimed to prove that both position estimation and BMF values can be solved simultaneously, by using data from two adjacent magnetometers, fixed close (22 mm) to each other. A drawing illustrating the concept of our sensor is presented in Figure 1.

As the capabilities of MEMS magnetometers increase, so does the amount of new research in biomedical and general engineering fields regarding magnetic tracking. A multi-magnetometer system designed for eye tracking was described in [12]. A finger-tracking method was developed [13] for pointing input as a means for interfacing with electronic devices. An implementation of larger-scale magnetic tracking aimed at surgical tool guidance was presented in [14]. A very large cubic array of sensors was developed for capsule endoscope tracking by an active research group in the field [15]. Moreover, a thorough review of such systems was given in [16]. However, it should be noted that all research we were able to find used a much greater number of magnetometers than just two, as proposed in this paper.

## 2. Materials and Methods

### 2.1. Linear Position Calculation

A thorough description of the magnetic position determination method can be found in our previous work [8]. However, for readability, the model and basic position calculation principle are briefly explained here. Formula (1) is the model describing the *B* field value of a cylindrical magnet at point X [17].

(1) $$B=B_{T}\left(\frac{3({\hat{m}}_{u}\cdot X)X}{R^{5}}-\frac{{\hat{m}}_{u}}{R^{3}}\right),$$

(2) $$B_{T}=\left(\mu_{r}\mu_{0}\pi r^{2}LM_{0}\right)/\left(4\pi \right),$$

(3) X=((x−a),(y−b),(z−c)),

(4) $$R=\sqrt{\left({\left(x-a\right)}^{2}+{\left(y-b\right)}^{2}+{\left(z-c\right)}^{2}\right)},$$

Here, ${\hat{m}}_{u}=\left(m,n,p\right)$ is a normalized vector of the dipole’s magnetic moment, representing the orientation of the magnet’s magnetism, (m,n,p) being the vector’s projections to the (*x*, *y*, *z*) axes, respectively. $B_{T}$ is a constant calculated by Formula (2), where $\mu_{r}$ is the relative permeability of the medium (air), $\mu_{0}=4\pi \times 10^{-7}$ (T·m/A) is the magnetic constant, L the length of the magnet (m), r the radius of the magnet (m), and $M_{0}$ the magnetization of the magnet (A/m). × is the location of a magnetometer found by (3), in relation to the permanent magnet location, which is (a,b,c)=(0,0,0) and represents the main frame of reference. *R* is the magnitude of the × vector calculated by Formula (4) [17]. It should be mentioned that the expressions (1)–(4) describe a first-order dipole approximation, which is applicable (with a maximum error of less than 1%) to points no closer than 1.5 $\times \sqrt{R^{2}+{\left(L/2\right)}^{2}}$ to the magnet’s center [18]. In our case of a 2 × 2 mm cylindrical magnet, it would be 2.14 mm.

The model (1) can be exploded into three separate equations for each axial constituent of the magnetic field (5)–(7) [17].

(5) $$B_{x}=B_{T}\left(\frac{3[m(x-a)+n(y-b)+p(z-c)](x-a)}{R^{5}}-\frac{m}{R^{3}}\right),$$

(6) $$B_{y}=B_{T}\left(\frac{3[m(x-a)+n(y-b)+p(z-c)](y-b)}{R^{5}}-\frac{n}{R^{3}}\right),$$

(7) $$B_{z}=B_{T}\left(\frac{3[m(x-a)+n(y-b)+p(z-c)](z-c)}{R^{5}}-\frac{p}{R^{3}}\right).$$

The position calculation is performed by using the least-squares error (LSE) method. It solves the provided function (equation system) by gradiently changing the values of an initial guess, until the squared difference between the function inputs and solutions is minimal. Biancalana et al. [12] gave a strong discussion about the importance of the initial guess in the optimization algorithm. In our case, it is helpful that the whole measurement takes place in strictly one hemisphere of the permanent magnet’s magnetic field, removing the possibility for B values to duplicate throughout the range of the measurement. The range in the algorithm is restricted by using spatial boundaries. We chose an initial guess near zero of the dynamic range (maximum B field) for the first position and used the previous position as an initial guess for the next one, which reduced the computation time.

### 2.2. Background Magnetic Field Calculation

Simply subtracting external reference B values from the main B values [8] is a primitive approach, which requires a reference sensor to be outside of the magnetic field of the permanent magnet. We raised the hypothesis that two adjacent magnetometers could be used for both position and BMF calculation. However, the position of the reference magnetometer (in relation to the main magnetometer) must be known and constant. In such a case, both sensors could be used inside of the magnetic field of the permanent magnet, which would significantly reduce the size of the system.

By adding 3 extra measured values $\left(B_{refx},B_{refy},B_{refz}\right)$ with 3 extra equations describing them, 3 more unknown variables could be solved. In our case, in addition to *x*, *y*, and *z* position determination, such an algorithm could solve 3 constituents of the BMF $\left(BMF_{x},BMF_{y},BMF_{z}\right)$, while simultaneously compensating for them. The system of equations for the improved model is presented below, where B values are in (8)–(10) for the main magnetometer and $B_{ref}$ values are in (13)–(15) for the reference magnetometer. The connection between the main and the reference equations is described in Equations (11)–(14).

(8) $$B_{x}=B_{T}\left(\frac{3[m(x-a)+n(y-b)+p(z-c)](x-a)}{R^{5}}-\frac{m}{R^{3}}\right)+BMF_{x},$$

(9) $$B_{y}=B_{T}\left(\frac{3[m(x-a)+n(y-b)+p(z-c)](y-b)}{R^{5}}-\frac{n}{R^{3}}\right)+BMF_{y},$$

(10) $$B_{z}=B_{T}\left(\frac{3[m(x-a)+n(y-b)+p(z-c)](z-c)}{R^{5}}-\frac{p}{R^{3}}\right)+BMF_{z},$$

(11) $$x_{ref}=x,$$

(12) $$y_{ref}=y,$$

(13) $$z_{ref}=z+\Delta z,$$

(14) $$R_{ref}=\sqrt{({\left(x_{ref}-a\right)}^{2}+{\left(y_{ref}-b\right)}^{2}+{\left(z_{ref}-c\right)}^{2}),}$$

(15) $$B_{refx}=B_{T}\left(\frac{3\left[m\left(x_{ref}-a\right)+n\left(y_{ref}-b\right)+p\left(z_{ref}-c\right)\right]\left(x_{ref}-a\right)}{R_{ref}^{5}}-\frac{m}{R_{ref}^{3}}\right)+BMF_{x},$$

(16) $$B_{refy}=B_{T}\left(\frac{3\left[m\left(x_{ref}-a\right)+n\left(y_{ref}-b\right)+p\left(z_{ref}-c\right)\right]\left(y_{ref}-b\right)}{R_{ref}^{5}}-\frac{n}{R_{ref}^{3}}\right)+BMF_{y},$$

(17) $$B_{refz}=B_{T}\left(\frac{3\left[m\left(x_{ref}-a\right)+n\left(y_{ref}-b\right)+p\left(z_{ref}-c\right)\right]\left(z_{ref}-c\right)}{R_{ref}^{5}}-\frac{p}{R_{ref}^{3}}\right)+BMF_{z}.$$

Here, Δz is the distance between the magnetometers (on the sensor PCB board) in the *z* axis. On the other axes, the main-to-reference magnetometer distances Δx and Δy are equal to 0, but could be added as well, if needed.

### 2.3. Predicting Jaw Rotation from Sensor-Based Translation Estimate

Single-magnetometer-based position estimation is possible if the orientations of the magnetometer and the magnet are known. This would suggest that it is only possible to evaluate the relative 3D position of two objects that are unable to rotate in any axis, resulting in 3-DOF position estimation. However, if rotation correlates with translation in a rational way, it should be possible to assess probable sensor angle values from the sensor-based 3-DOF position estimate. Therefore, we raised the hypothesis that if the rotation of the human jaw correlates with translation, it should be possible to implement an algorithm estimating the 5-DOF position from 3-DOF data.

The temporomandibular joint can both rotate and slide forward. The forward sliding trajectory (condylar path) is dependent on the size of the articular tubercle, otherwise called the articular eminence. Some people have almost a straight horizontal condylar path, while others’ jaws slide forward and slightly downward onto the articular tubercle [19]. Vertical rotation around the *x* axis (opening): When the jaw opens first 10–20°, it only rotates. When opening further, it starts sliding forward, with both joints follow the condylar path. It also continues rotating. In chewing, talking, or bruxing, the jaw usually does not reach such an angle. Horizontal (side) rotation around the *y* axis: When the jaw turns to the side, it rotates around the joint of that side to which it is turning, while the joint of the other side slides forward, following the condylar path. The only rotation around the *z* axis is the slight tilting during the side (*y*) rotation—while one joint is fixed translationwise and the other side slides onto the articular tubercle. Otherwise, the jaw is not able to rotate around the *z* axis, meaning you cannot open only one side of the jaw while the other remains in contact.

However, predicting jaw rotation around the *z* axis is not possible—its rotation cannot be linked to any translation, as the jaw is free to translate (slide) along the *z* axis, following the condylar path. With said reasons in mind, the z rotation was not accounted for in our method. Thankfully, the slight *z* axis tilting during side (*y* axis) turn has a trivial effect on the position evaluation, compared to the significant advantages of predicting the other two DOFs of rotation (*x* and *y*).

**Figure 2:**
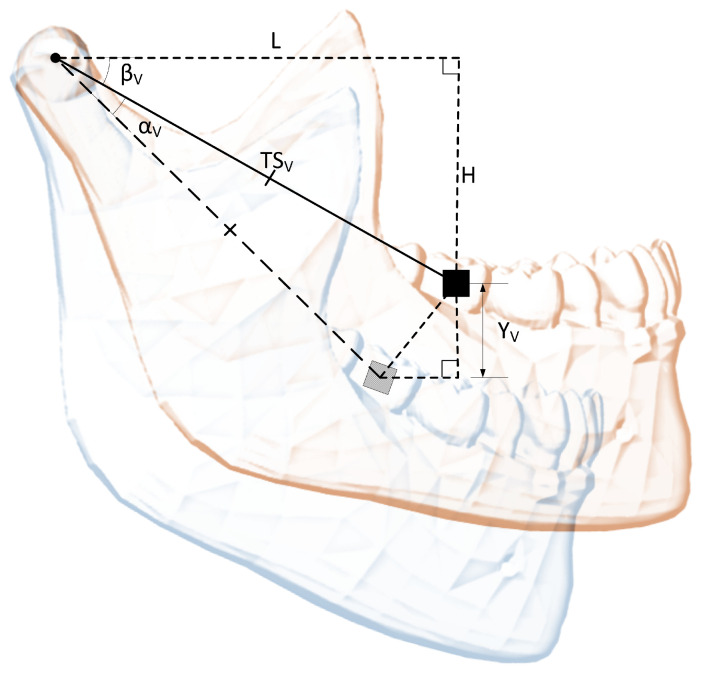
Vertical rotation angle relation to vertical linear displacement. Based on [20].

Vertical rotation (around the *x* axis) angle $\alpha_{V}$ can be linked to vertical translation $Y_{V}$ via (18)–(20), with an explanatory drawing presented in Figure 2. The *L* (length) and *H* (height) dimensions must be measured, while $Y_{V}$ is calculated from sensor output.

(18) $$TS_{V}=\sqrt{L^{2}+H^{2}},$$

(19) $$\beta_{V}=arccos\left(\frac{L}{TS_{V}}\right),$$

(20) $$\alpha_{V}=arcsin\left(\frac{H+Y_{V}}{TS_{V}}\right)-\beta_{V}.$$

**Figure 3:**
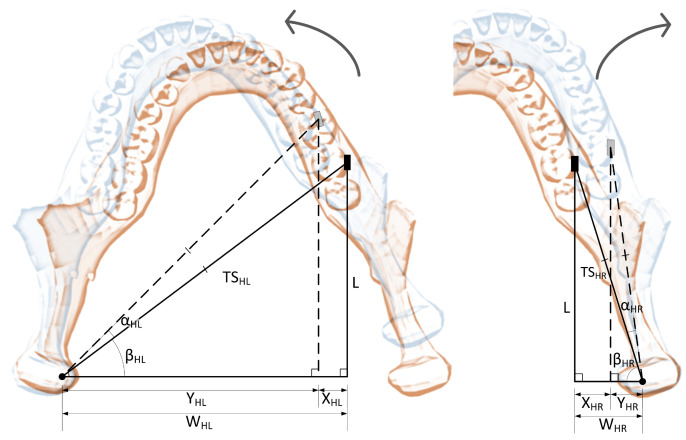
Horizontal rotation angle relation to left and right horizontal linear displacements. Based on [20].

Linking horizontal rotation (around *y* axis) angle $\alpha_{H}$ to horizontal (side) translation $X_{H}$ is slightly more complicated. Since the sensor is placed on one side of the jaw, the dimensions are different for each side, although the same Equations (21)–(24) are applicable for both cases. An explanatory drawing for horizontal rotations is presented in Figure 3. The *L* (length) and $W_{H}$ (width) dimensions must be measured, while $X_{H}$ is calculated from sensor output.

(21) $$TS_{H}=\sqrt{L^{2}+W_{H}^{2}},$$

(22) $$Y_{H}=W_{H}-X_{H},$$

(23) $$\beta_{H}=arcsin\left(\frac{L}{TS_{H}}\right),$$

(24) $$\alpha_{H}=arccos\left(\frac{Y_{H}}{TS_{H}}\right)-\beta_{H}.$$

Rotation of the sensor coordinate system centered around the main magnetometer calls to re-evaluate the change of the reference magnetometer position. If the sensor did not rotate, there would be a constant 22 mm displacement at the *z* axis (as placed in the PCB) and 0 mm at *x* and *y* axes. However, when rotations are involved, projection on the *z* axis shortens, and projections on the *x* and *y* axes increase. Rotation in two axes results in translation in all three axes. This is described in Equations (25)–(28), taking into account that the limits of the jaw rotation are far less than 90°:

(25) $$y_{ref}=y+\left(\Delta z\cdot sin\left(\alpha_{V}\right)\right),$$

(26) $$\Delta z_{y}=\Delta z\cdot cos\left(\alpha_{V}\right),$$

(27) $$x_{ref}=x+(\Delta z_{y}\cdot sin\left(\alpha_{H}\right)),$$

(28) $$z_{ref}=\Delta z_{y}\cdot cos\left(\alpha_{H}\right).$$

It should be noted that based on the orientation of the skull’s coordinate system specified in Figure 1, the only possible translation in the *y* (vertical) axis is negative. Negative translations in other axes are matched by negative rotation angles as well. In practice, 5-DOF (extra 2 DOFs) evaluation was implemented by adding trigonometric translation-to-rotation dependency equations to the optimization algorithm. During each iteration of the optimization algorithm (even an initial guess), we rotated the calculated B values using a rotation matrix, by the angles acquired from the translation-to-rotation dependency equations. Only then, the result was compared to the measured B values.

### 2.4. Sensor Prototype

**Figure 4:**
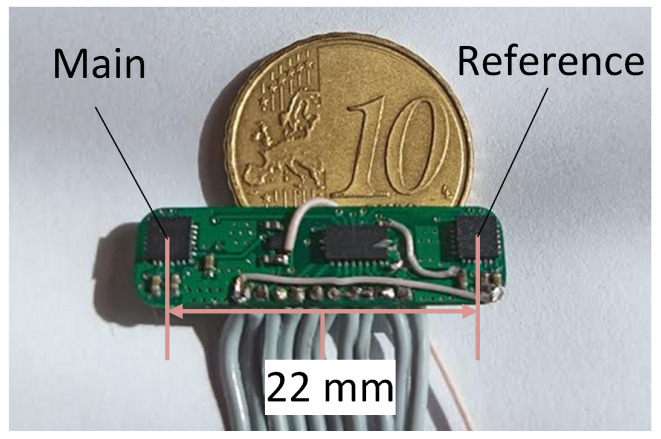
Prototype sensor board.

A sensor prototype board was created, with dimensions of (26.3 × 5.5 × 1) mm. It is presented in Figure 4. Its main components were two ICM-20948 (TDK InvenSense, San Jose, CA, USA) inertial measurement units (IMUs) containing 3-axis magnetometers, placed 22 mm apart (center-to-center). The sensor was controlled externally using I2C communication.

### 2.5. 5-DOF Method Experiments

**Figure 5:**
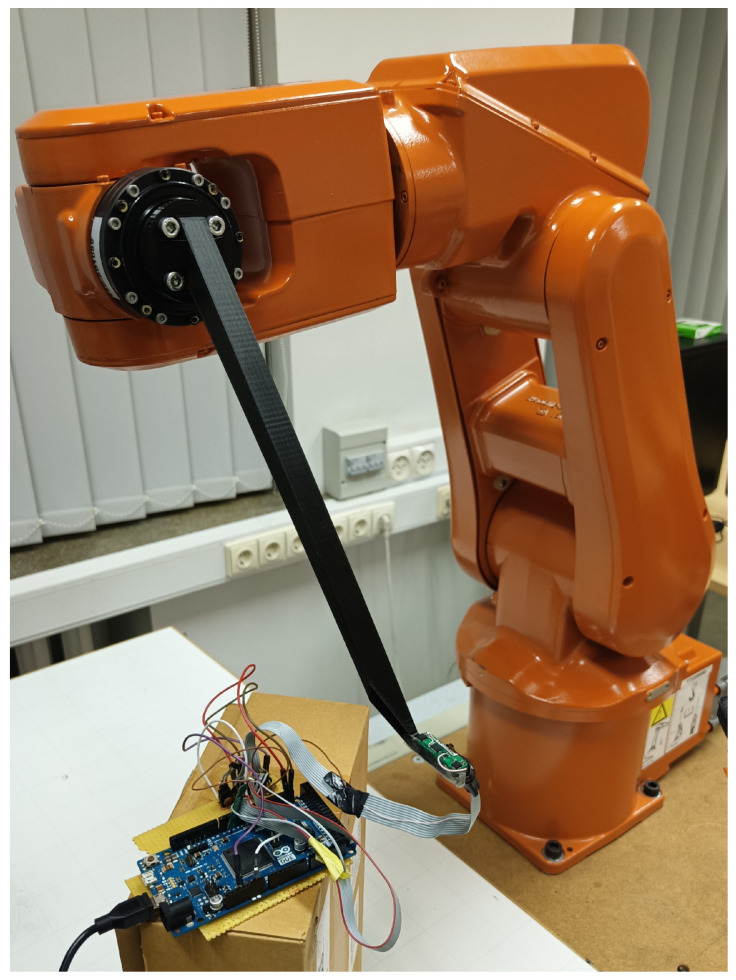
Robotic arm with the sensor fastened to the custom mounting tool.

**Figure 6:**
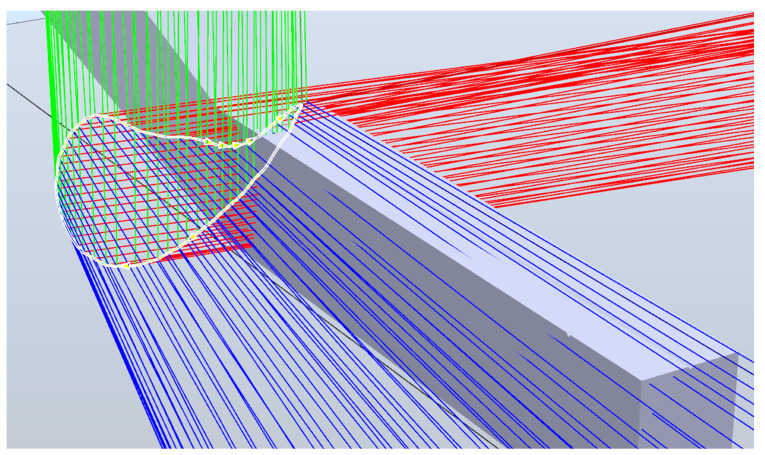
Programmed masticatory trajectory path in the RobotStudio software.

The 3-DOF test trajectory was expanded to 5 DOFs, using jaw angle-to-linear displacement Equations (18)–(24) described above. To move the sensor prototype in the 5-DOF test trajectory, the IRB120-3/0.6 (ABB, Zurich, Switzerland) robotic arm was used. The robot and programmed trajectory can be seen in Figure 5 and Figure 6, respectively. Red, green, and blue lines represent the *x*, *y*, and *z* of the origin of each point in the trajectory. The specifications of the robotic system state 0.01 mm linear and 0.01° angular repeatability. However, the robotic arm has motors that contain both permanent magnets and electromagnets. Fortunately, our proposed BMF compensation method is able to compensate for any BMFs that affect both magnetometers equally. Therefore, it does not matter if it is the natural magnetic field of the Earth or one generated by an industrial robot, as long as it is homogenous in the volume of the sensor. Therefore, a custom sensor-mounting tool was 3D printed, to fasten the sensor further from the robot, so its magnetic field would be weaker and more homogenous in the volume of the sensor. The trajectory was first run without a permanent magnet in the vicinity, to assess the homogeneity of the ambient field (in the volume of the sensor), by comparing the data of the magnetometers. The average differences were ($\Delta B_{x}$, $\Delta B_{y}$, $\Delta B_{z}$) = (0.99, 3.27, 0.59) µT. These differences could be described as offsets, because they remained constant during robot motion and B value changes. In such a manner, the sensor was moved away from the flange of the robot by 18 cm in the *Y* (vertical) and by 16 cm in the *Z* (perpendicular) axes. While effectively mitigating the effects of the robot’s magnetic field, such a tool certainly increased the positioning uncertainty by slightly bending and vibrating while in motion.

For the laboratory experiments, the trajectory representing natural masticatory motion was sampled from [21]. This particular trajectory was chosen for consistency, with the purpose of comparing the results with our previous work, where it was already used. The data were recorded at a 60 Sa/s rate. For static position testing, the robotic arm stopped at each point of the trajectory, while 20 measurements’ average value was recorded. The aim of this test was to evaluate the ability of the algorithm itself, hence the stopping and the averaging to obtain precise B values and to overcome the limitations of the magnetometers’ noise and data collection frequency. For dynamic testing, the masticatory trajectory was recreated with the maximum speed of the robot, taking 2 s to complete one cycle. To smooth the estimated trajectory, a moving average filter was used with a window size of N = 4. The aim of this test was to evaluate the method in action, taking into account the limitations of the magnetometers’ noise and data collection frequency.

## 3. Results

### 3.1. Background Magnetic Field Calculation

**Figure 7:**
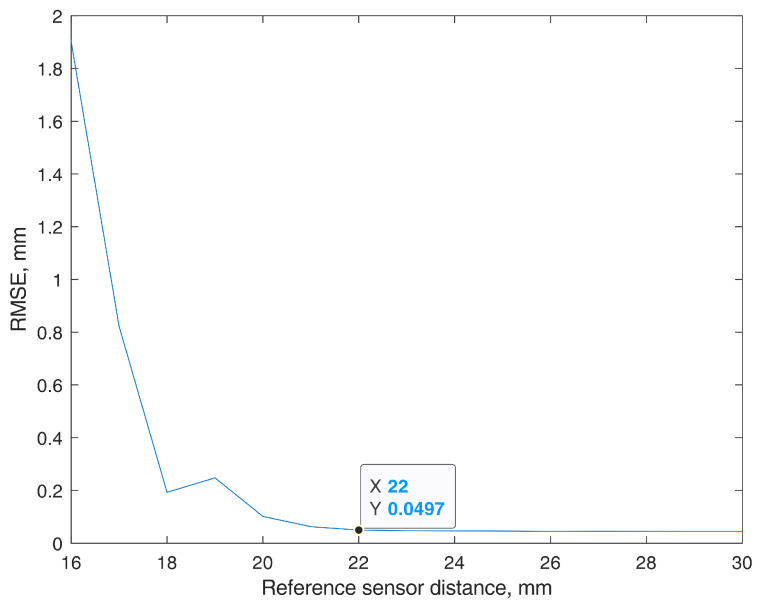
Dependency between the RMSE (Root mean square error) and reference sensor distance to the main sensor. Finite element model data.

Based on proposed position and BMF determination methodology, an experimental algorithm was created. It was able to determine the BMF values and estimate instantly the BMF-compensated position of the main sensor. The proposed method for BMF compensation was tested using the finite element model of a permanent magnet. Magnetic field values were calculated throughout the space (100 × 100 × 100 mm) surrounding the magnet, with a step of 0.1 mm. The modeled space was evenly contaminated with an upward 65 μT vector, BMF = [0 65 μT 0]. Magnetic field values of two identical trajectories were extracted, representing two moving magnetometers with a constant distance from each other. These data were used to estimate the trajectory of the main sensor. To determine the optimal distance between the main and the reference magnetometers, the sensors were tested in the finite element model. Trajectory estimates by theoretical sensors, with reference magnetometer distances varying from 10 mm to 30 mm, were compared to the original trajectory. This resulted in the RMSE-to-distance dependency presented in Figure 7. We chose the minimal distance at which the errors stopped significantly decreasing. Therefore, the experimental sensor prototype was manufactured with a 22 mm center-to-center distance between the magnetometers.

**Figure 8:**
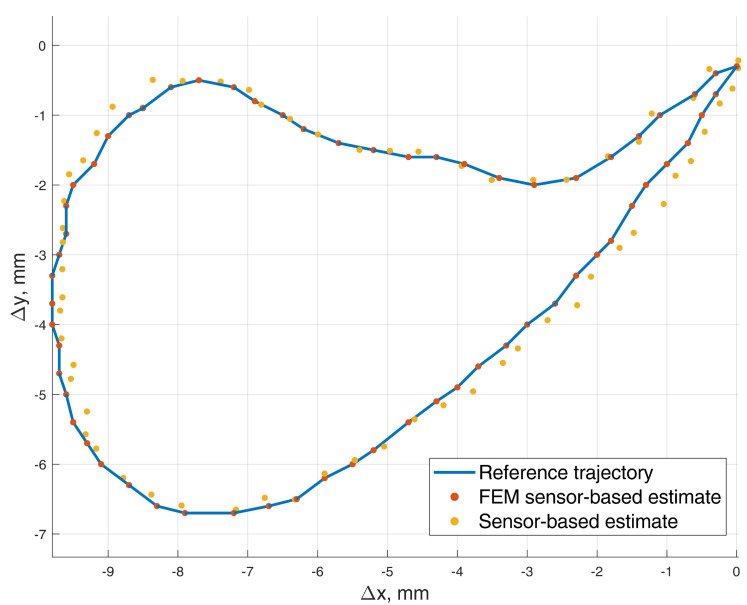
Original input trajectory and determined trajectory while using BMF (Background magnetic field) compensation. Finite element model simulation, RMSE = 0.05 mm (red), and prototype sensor–based data with real BMF, RMSE = 0.28 mm (yellow).

In Figure 8, the original trajectory is presented, along with the positions estimated using the magnetic method with BMF compensation, both in the finite element model and laboratory environments. The FEM sensor-based 3-DOF trajectory is presented in red, with an RMSE of 0.05 mm. The determined BMF vector was equal to BMF = [−7.10 μT 6.87 μT −0.05 μT]. For comparison, the single-magnetometer method with data not even contaminated by the BMF showed an RMSE of 0.1 mm in the same finite element environment. The trajectory estimated during the analogous 3-DOF experiment in the real world, with the natural BMF and using our sensor prototype, is presented in yellow. The test was controlled by a 3D positioning system, EMS 301 (Elintos Matavimo Sistemos, Kaunas, Lithuania), stated to have 0.1 mm step positioning resolution. The experiment resulted in an RMSE of 0.28 mm. The determined BMF vector was equal to BMF = [−8.95 μT 57.19 μT −12.24 μT]. For comparison, our previously proposed [8] single-magnetometer method, with pre-measured and subtracted BMF components, showed an RMSE of 0.26 mm on the same equipment.

### 3.2. Jaw Angle Calculation

**Figure 9:**
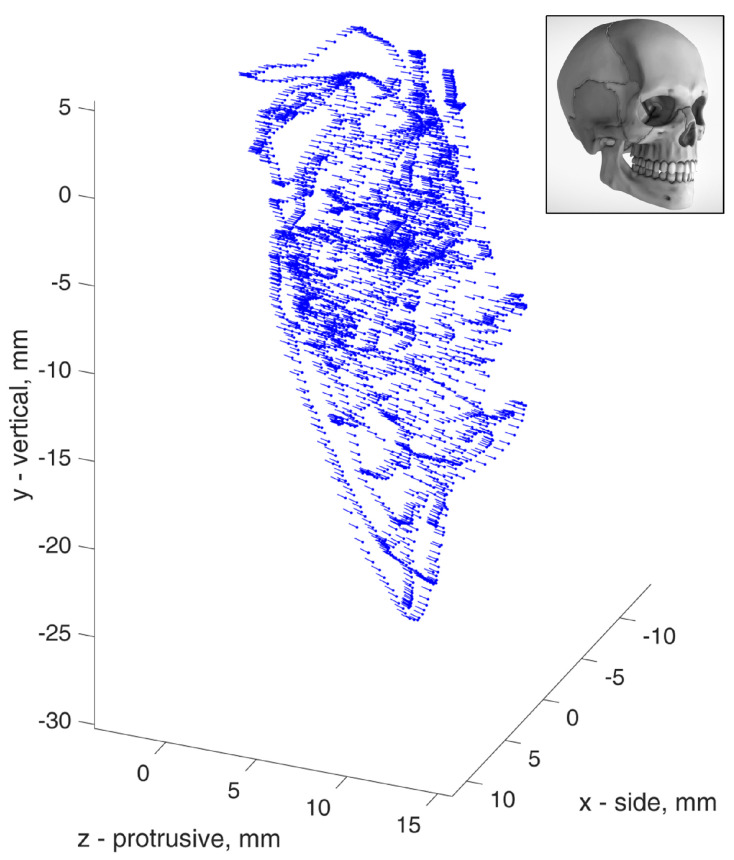
Cloud of recorded 6–DOF (Degree of freedom) reference jaw positions with rotational angles, depicted as vector arrows. The skull image [11] is provided as the orientation reference.

To demonstrate the validity of jaw angle equations, the calculation results for one volunteer were compared to reference data acquired from the same person, using the TrakStar 3D (Ascension Technology Corporation, Shelburne, VT, USA) position tracking system. The reference data were a cloud of 6-DOF points, as presented in Figure 9, recorded while the volunteer moved the jaw to many various positions.

**Figure 10:**
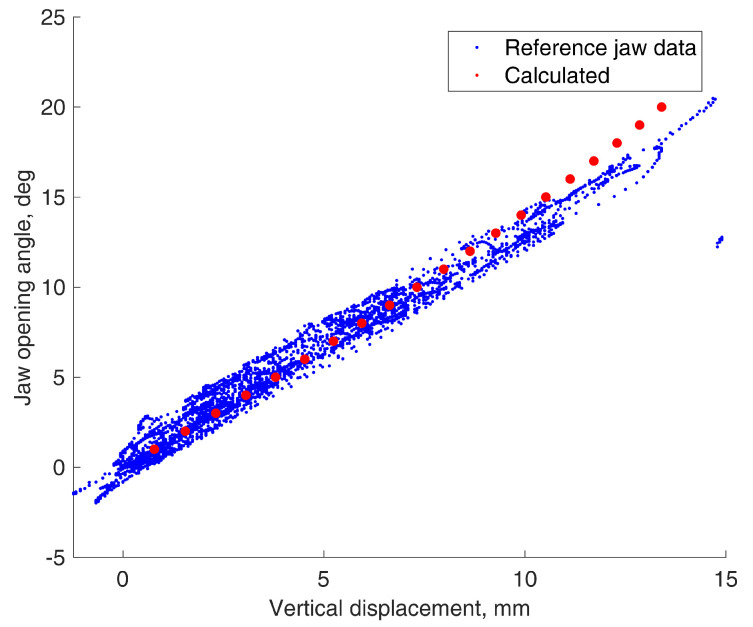
Vertical translation to jaw opening rotation dependencies.

**Figure 11:**
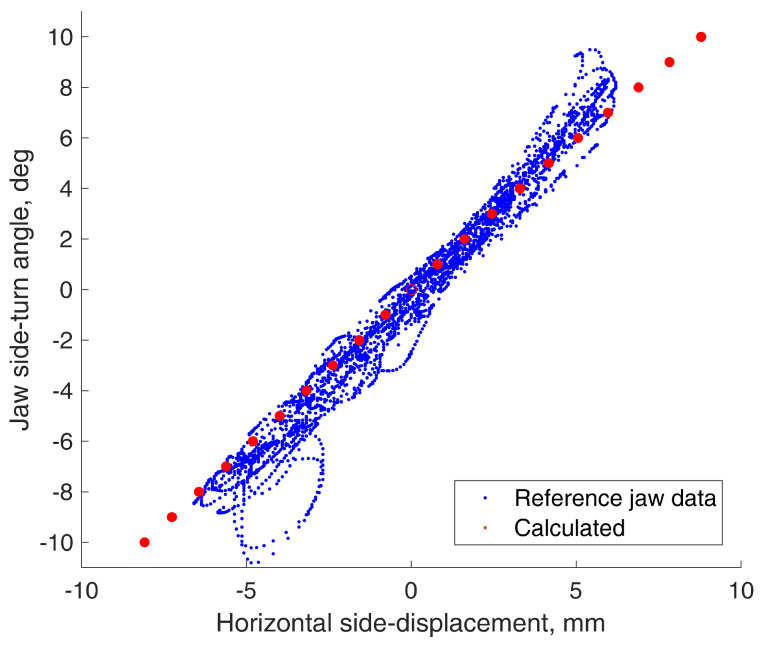
Horizontal (side) translation to jaw side rotation dependencies.

Points representing rotation-to-translation dependencies were plotted from every reference data point and compared to calculations based on the proposed equations. The jaw parameters of the volunteer were measured by hand using a ruler and were equal to L = 45 mm, H = 33 mm, $W_{L}$ = 64 mm, and $W_{R}$ = 16 mm. The comparisons are presented in Figure 10 and Figure 11.

As we can see on this particular jaw, the rotation-to-translation dependency seems nearly linear. From the trigonometrical perspective, we know that non-linearity increases with higher angle values, but the jaw cannot rotate much. The largest rotation angle is of the vertically opening jaw, and it is 35° at a maximum [22]. Moreover, in natural circumstances, we rarely fully open the jaw. This suggests the possibility to use simple coefficients in the future, instead of the proposed equations, to reduce the calculations in practical use. However, in this paper, we used the given equations.

### 3.3. 5-DOF Prototype Experiments

#### 3.3.1. Static Positioning Test

**Figure 12:**
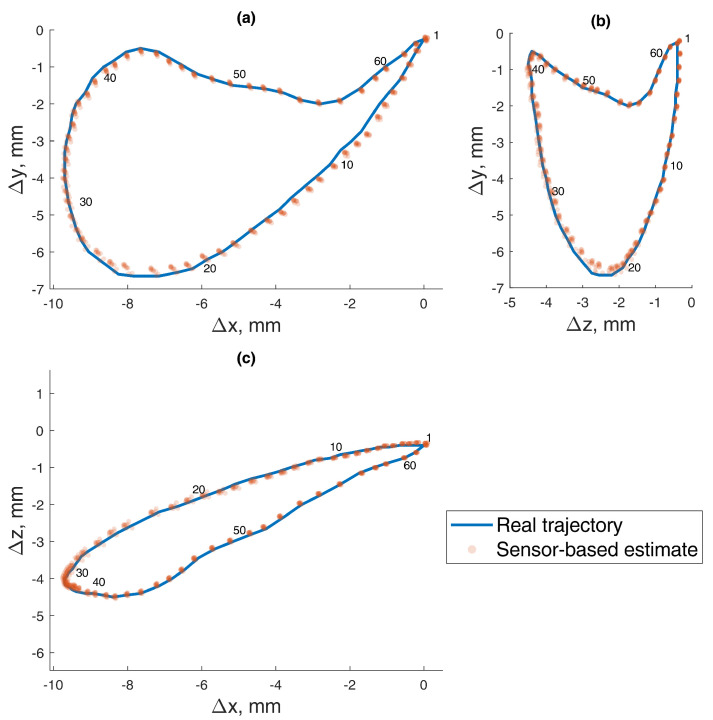
The 5–DOF masticatory test trajectory estimated with BMF compensation and angle compensation. Magnetic values at each point recorded with the sensor in the static state. Average RMSE = 0.17 mm, ED = 0.10 mm (Euclidian distance). Measured points are numbered 0 to 62, and all 10 iterations are represented in transparent markers. (**a**) Lateral–vertical view (*x*–*y*). (**b**) Protrusive–vertical view (*z*–*y*). (**c**) Lateral–protrusive view (*x*–*z*).

**Figure 13:**
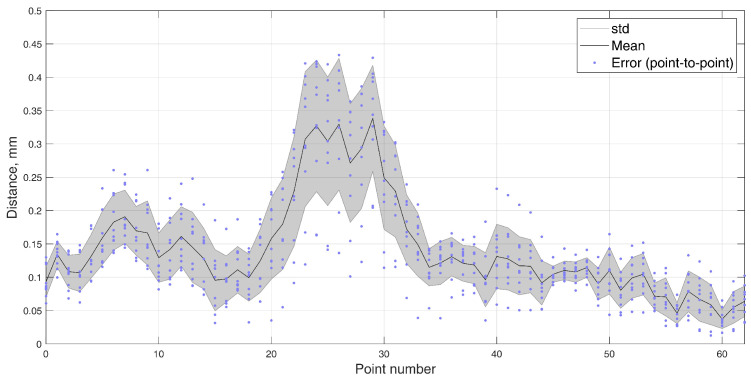
Error (point–to–point) of every measured point in 10 iterations of the masticatory test trajectory in the static positioning test, with the mean and standard deviation.

**Figure 14:**
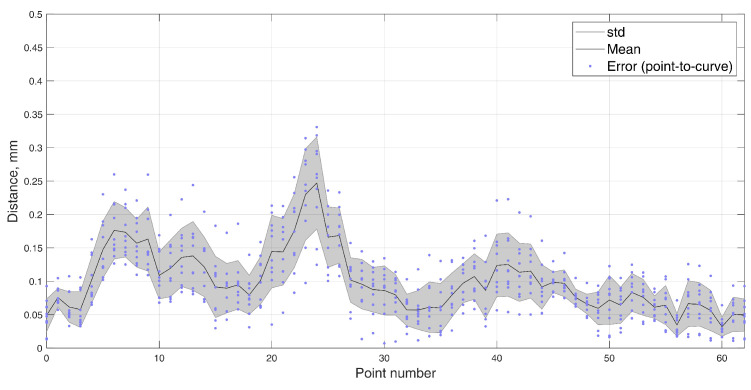
Error (point–to–curve) from the reference masticatory trajectory to the estimated trajectory in 10 iterations of the static positioning test, with the mean and standard deviation.

In this 5-DOF experiment, both the BMF and jaw angle compensation methods were used. In the precision experiment, the test masticatory trajectory was replicated with a robotic arm for 10 iterations, stopping for measurement at each point of the trajectory. The recorded positions are presented in Figure 12, with a mean RMSE and standard deviation (STD) of 0.165±0.020 mm. The distribution of the errors for each point is presented in Figure 13. For a comparison to the dynamic experiment, the mean Euclidean distance (ED) from the reference trajectory to the sensor-estimated trajectory was calculated and presented in Figure 14, resulting in a mean ED and STD of 0.098±0.014 mm.

#### 3.3.2. Dynamic Test

**Figure 15:**
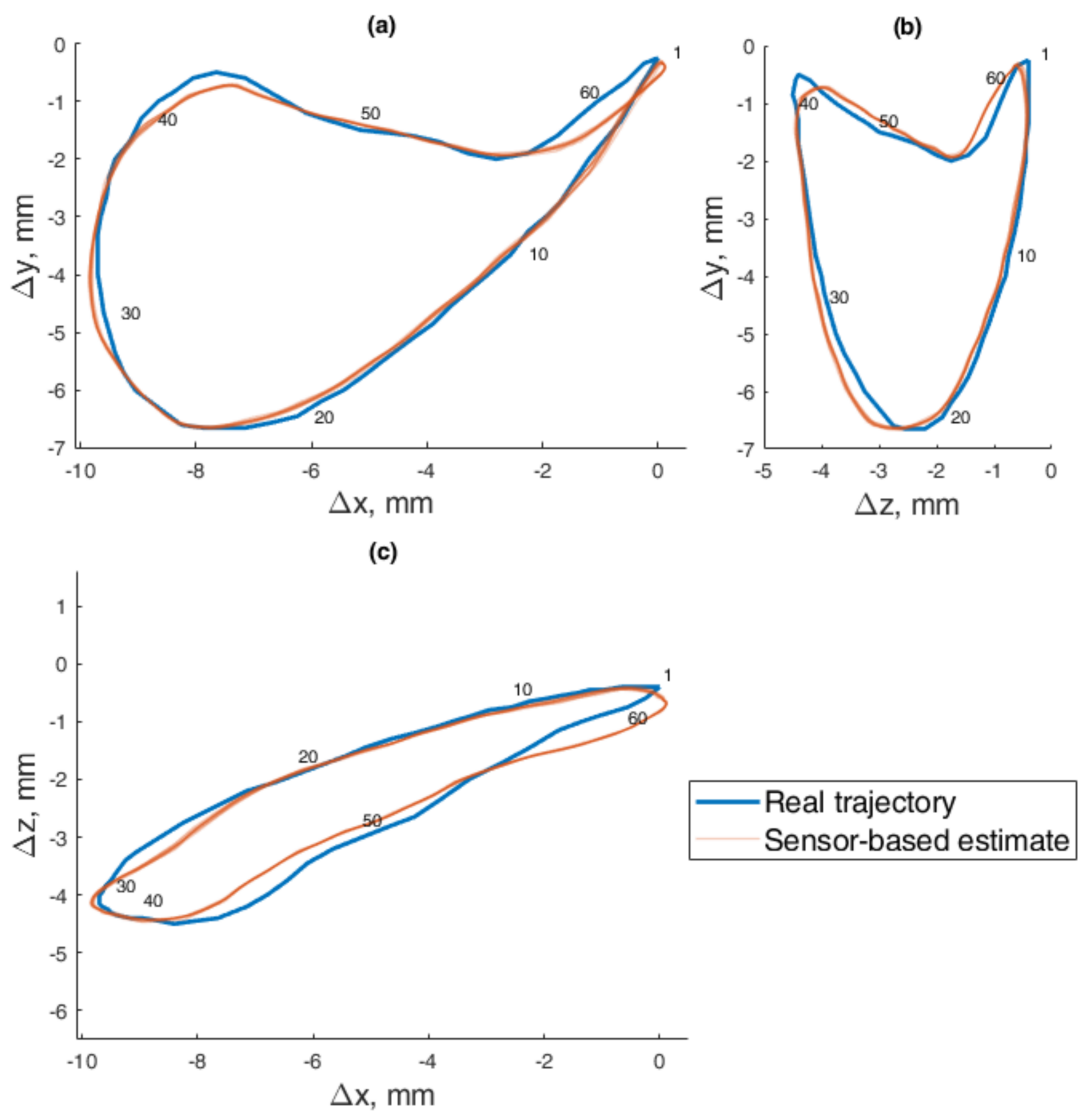
The 5–DOF masticatory test trajectory estimated with BMF compensation and angle compensation. Magnetic values recorded with the sensor in motion, 2 s per cycle. Average ED = 0.18 mm. Measured points are numbered 0 to 62, and all 10 iterations are represented in transparent markers. (**a**) Lateral–vertical view (*x*–*y*). (**b**) Protrusive–vertical view (*z*–*y*). (**c**) Lateral–protrusive view (*x*–*z*).

**Figure 16:**
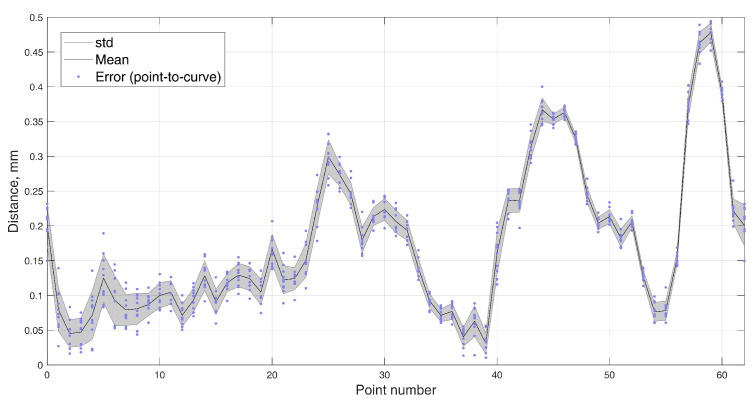
Error (point–to–curve) from the reference masticatory trajectory to the estimated trajectory in 10 iterations of the dynamic test, with the mean and standard deviation.

In the dynamic experiment, the test masticatory trajectory was recreated 10 times, with the maximum speed of the robot, taking 2 s per every cycle. Since the output of the magnetometers had noise fluctuations, data averaging was very effective at increasing the method’s precision, as was done in the static positioning experiment. In dynamic testing, it is not possible to stop during each measurement, so a moving average filter was used with a window size of four values. Such a small window size was chosen because data were recorded at ∼60 Sa/s rate, resulting in only ∼120 values per trajectory. The recorded trajectories are presented in Figure 15, and the mean ED and STD from the reference to estimated trajectory was 0.175±0.003 mm. The distribution of the errors for each point is presented in Figure 16.

It should be noted that a large influence on the shape discrepancies of the sensor-based trajectory estimate came from the elasticity and springiness of the long, plastic sensor-mounting tool, which showed a slight delay and amortization after sudden movements.

## 4. Conclusions and Discussion

**Figure 17:**
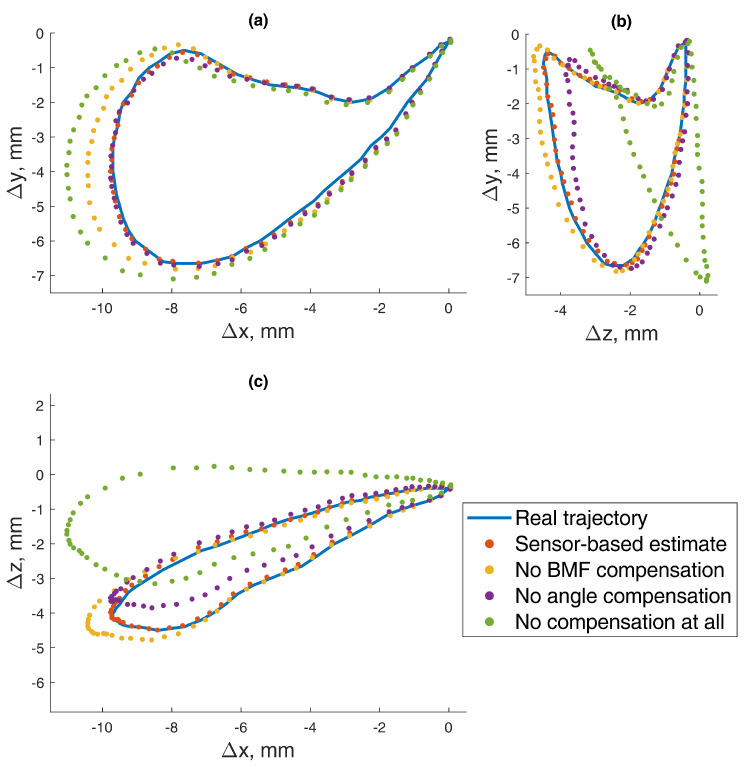
The 5–DOF masticatory test trajectory estimated with and without the BMF compensation and angle compensation algorithms. Magnetic values at each point recorded with the sensor in the static state. (**a**) Lateral–vertical view (*x*–*y*). (**b**) Protrusive–vertical view (*z*–*y*). (**c**) Lateral–protrusive view (*x*–*z*).

The idea of tracking jaw motion using a permanent magnet dates back to 1977 [23], although the technical limitations of that time predetermined that the method could work only for primitive motion recognition. In our previous work [8], we developed a method based on a modern three-axis magnetometer and were able to determine the position of a permanent magnet with an RMSE of 0.26 mm. This paper described solutions for two of the main drawbacks of the proposed magnetic jaw-position-tracking method. To illustrate the said drawbacks, in Figure 17, the data are presented with and without the algorithms proposed in this paper.

Rotation-to-translation equations could enable the practical implementation of the method, since previous precision testing was performed only in 3-DOF conditions. It was proven by trigonometric equations and reference data that it is possible to predict jaw rotation when its linear translation is known. We recognize that we simplified the complex dynamics of the jaw, for example by ignoring the influence of the articular tubercle curvature and excluding any rotation around the *z* axis from calculations. Be that as it may, the proposed equations fulfill their objective of predicting the jaw rotational angle moderately well, thus enabling a 3-DOF position determination method to be used on the 5-DOF human jaw.

The two-adjacent-magnetometer approach allows for any homogenous BMF compensation, while also improving the precision of the system, since measurements from both magnetometers are included in position determination. Moreover, it minimizes the dimensions of the system, in comparison to subtraction-based BMF compensation, making it suitable for intra-oral use. In the practical experiments, the conditions were unfavorable due to the significant magnetic fields generated by the robotic positioning system. However, the method displayed resistance to external magnetic interference, while achieving a mean RMSE and STD of 0.165±0.020 mm, a 37% decrease in error, compared to the single-magnetometer 3-DOF experiments with pre-compensated BMF. The dynamic test resulted in an ED and STD of 0.212±0.002 mm, which is a significant increase in error compared to the ED and STD = 0.098±0.014 mm of the static test. Dynamic measurements were slightly impeded by a low data acquisition frequency and the elasticity of the long sensor-mounting tool. Hence, in future developments, faster data acquisition and wireless data transmission should be the key to making the method practical and commercially plausible. A summary and comparison of available results from current and previous work [8] and error projections on different axes are presented in Table 1 and Table 2.

A significant drawback of the method is the offset drift of the magnetic sensors, which is especially triggered by stronger magnetic fields. As for the ICM20948 sensors that were used in our experiments, the drift may be up to ±30 μT, which may induce errors of up to several mm, which decrease when approaching the magnet. Therefore, at least with current technology, the offset of the magnetometers must be calibrated. In future studies, the sensor should be further minimized in size, equipped with a battery and wireless communication, and tested on volunteers after approval of the bio-ethics commission. To sum up, 5-DOF experiments on a precise robotic system confirmed both the BMF and angle compensations simultaneously, proving the method to be eligible for further development.

## Figures and Tables

**Table 1:** Mean and standard deviation in mm of the root-mean-squared error (RMSE) and its projections on different axes with current and previous approaches.

Approach, Test	RMSE	RMSE, *x* proj.	RMSE, *y* proj.	RMSE, *z* proj.
1 magnetometer, static	0.260±0.004	0.174±0.003	0.107±0.001	0.064±0.001
2 magnetometers, static	0.165±0.020	0.084±0.006	0.089±0.015	0.035±0.006

**Table 2:** Mean and standard deviation in mm of the Euclidean distance (ED) and its projections on different axes with current and previous approaches.

Approach, Test	ED	ED, *x* proj.	ED, *y* proj.	ED, *z* proj.
1 magnetometer, static	0.127±0.002	0.071±0.001	0.059±0.002	0.060±0.001
2 magnetometers, static	0.098±0.014	0.049±0.014	0.056±0.010	0.042±0.009
2 magnetometers, dynamic	0.212±0.002	0.124±0.001	0.071±0.001	0.146±0.002

## Data Availability

The data presented in this study are openly available in the FigShare repository at 10.6084/m9.figshare.17430482 (accessed on 24 December 2021).

## Abbreviations

The following abbreviations are used in this manuscript:

BMF	Background magnetic field
DOF	Degree of freedom
ED	Euclidean distance
FEM	Finite element model
RMSE	Root-mean-squared error
STD	Standard deviation
